# Immunological Features of the Non-Structural Proteins of Porcine Reproductive and Respiratory Syndrome Virus

**DOI:** 10.3390/v7030873

**Published:** 2015-02-24

**Authors:** Edgar Rascón-Castelo, Alexel Burgara-Estrella, Enric Mateu, Jesús Hernández

**Affiliations:** 1 Laboratorio de Inmunología, Centro de Investigación en Alimentación y Desarrollo A.C (CIAD) Carretera a la Victoria Km 0.6, C.P. 83304 Hermosillo, Sonora, Mexico; E-Mails: erascon83@gmail.com (E.R.-C.); alexel.burgara@gmail.com (A.B.-E.); 2 Centre de Recerca en Sanitat Animal (CReSA), UAB-IRTA, Campus de la Universitat Autònoma de Barcelona, 08193 Bellaterra, Barcelona, Spain; E-Mail: enric.mateu@uab.cat; 3 Departament de Sanitat i d’Anatomia Animals, Universitat Autònoma de Barcelona, 08193 Bellaterra, Barcelona, Spain

**Keywords:** PRRSV, non-structural proteins, immune response

## Abstract

Porcine reproductive and respiratory syndrome virus (PRRSV) is currently one of the most important viruses affecting the swine industry worldwide. Despite the large number of papers published each year, the participation of non-structural proteins (nsps) in the immune response is not completely clear. nsps have been involved in the host innate immune response, specifically, nsp1α/β, nsp2, nsp4 and nsp11 have been associated with the immunomodulation capability of the virus. To date, only participation by nsp1, nsp2, nsp4 and nsp7 in the humoral immune response has been reported, with the role of other nsps being overlooked. Furthermore, nsp1, nsp2, nsp5, nsp7 nsp9, nsp10, nsp11 have been implicated in the induction of IFN-γ and probably in the development of the cell-mediated immune response. This review discusses recent reports involving the participation of nsps in the modulation of the innate immune response and their role in the induction of both the humoral and cellular immune responses.

## 1. Introduction

Porcine reproductive and respiratory syndrome (PRRS) was first described in the USA in 1987 [1] and appeared in Europe soon thereafter [2]. The causal agent, PRRS virus (PRRSV), was initially identified in Europe [2] and reported one year later in the USA [3]. Since then, PRRSV has become one of the pathogens that has the greatest impact on the swine industry, causing losses in the USA estimated at approximately 664 million US dollars per year [4].

**Figure 1:**
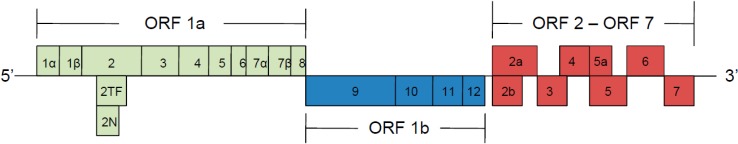
Porcine reproductive and respiratory syndrome virus (PRRSV) genome organization. ORF1a and 1b are translated into pp1a and pp1ab, which after proteolytic processing produce at least 14 nsps. ORF2 to ORF7 encode structural proteins associated with virion infectivity.

PRRSV is a single-stranded, positive-sense RNA enveloped virus that belongs to the *Arteriviridae* family, together with equine arteritis virus (EAV), lactate dehydrogenase-elevating virus (LDV) and simian hemorrhagic fever virus (SHFV) [5]. The viral genome is 15 kb and contains at least 10 open reading frames (ORFs) flanked by two untranslated regions 5’ and 3’. ORFs 1a and 1b, account for 75% of the viral genome and encode two long polypeptides (pp), pp1a and pp1ab; after enzymatic cleavage, these pp produce 14 non-structural proteins (nsps) that are implicated in viral replication, and two additional viral proteins called nsp2TF and nsp2N that results from ribosomal frameshifting [6,7]. The proteolytic process leading to the cleavage of nsps is performed by four viral proteases, encoded in the nsp1α, nsp1β, nsp2 and nsp4 regions. ORF2 to ORF7 are located at the 3’ terminus and code for eight structural proteins designated as GP2, E, GP3, GP4, GP5, ORF5a protein, M and N (Figure 1) [8,9,10,11].

Two genotypes of PRRSV have been described thus far: genotype 1, also known as the European type; and genotype 2, the North American type [12]. Both genotype 1 and 2 PRRSV infect cells of the monocyte/macrophage lineage. Homology between the genotypes varies depending on the gene or the protein that is being compared. Overall (for both genotypes), among the non-structural proteins, nsp1 and nsp2 are the most variable parts of the virus, with an identity of 50.5% to 54.3% and 24.4% to 28.0%, respectively; between the genotypes, nsp9 shows the highest conservation, with an identity of 73.2% to 75.0%. The most conserved structural protein is M protein (75.2% to 81.6% identity) [13].

PRRSV nsps are the first proteins to be expressed after infection and perform essential roles in viral replication (nsp9 to nsp12), with some being associated with virulence (nsp3 to nsp8) [14]. Recent studies have revealed the importance of nsps in the modulation of the immune response of pigs infected with PRRSV, but little information is available about the immune response elicited against these proteins. Here, we discuss recent reports involving the role of nsps in the modulation of the innate immune response as an evasion strategy of PRRSV and the possible role of nsps as a target for a protective immune response.

## 2. Non-Structural Proteins (nsps)

Fang and Snijder (2010) published an excellent review on the biological functions of the non-structural proteins of PRRSV that can be consulted for in-depth information [15]. The following is a brief summary of the roles of PRRSV nsps.

nsp1 is a 383-amino acid, multifunctional protein located at the amino terminus of pp1a and contains two subunits: nsp1α and nsp1β. Although nsp1α appears to be relatively conserved, nsp1β is highly variable [13]. Cleavage of nsp1 is autocatalytic and is thought to occur at Met or His 180 (depending on the genotype) [16]. The papain-like protease activity of nsp1α resides mainly in residues Cys 76 and His 146 or 147, whilst the papain-like protease activity of nsp1β resides in Cys 276 and His 345 [17]. Due to its zinc finger motifs, the participation of nsp1 is essential for the transcription of subgenomic mRNAs that are produced during the replication of arteriviruses, and certain point mutations in this protein can block the replication of PRRSV [18,19]. Kroese *et al.* (2008) mentioned that the loss of the papain-like-protease activity of nsp1α is related to the inhibition of sg mRNA synthesis, though the replication of genomic RNA is not affected. In addition, the loss of the papain-like-protease activity of nsp1β results in the non-processing of the nsp1β/nsp2 junction *in vitro*; therefore, no signs of viral RNA synthesis is detected [17].

nsp2 is the most variable of the PRRSV nsps, particularly the central region of nsp2. Several deletions have been reported in both genotype 1 [13,20,21] and genotype 2 strains [22,23,24], suggesting that nsp2 possesses functions non-essential for replication but that could be important/essential for nsp2’s function in host immunity. This protein has four functional regions: an NH_2_-terminus with a papain-like proteinase domain (PLP2) [25]; a non-specific central functional region, which differs in size and amino acid composition between PRRSV isolates; a hydrophobic region at the C-terminus and a conserved C-terminal tail [26,27]. nsp2 is produced in large quantities by infected cells and, together with nsp3 and nsp5, modifies the membrane of infected cells, favoring the assembly of the multi-protein complex of viral replication. In addition, nsp2 serves as cofactor for the function of nsp4 [28,29]. Recently, it has been shown that nsp2 may be associated with the virion [30], a fact that could explain why antibodies against nsp2 are immunodominant in infected pigs [31,32].

nsp4 is a chymotrypsin-like serine proteinase that is considered to be the most important virus protease since it contains a catalytic triad (His 1103, Asp 1129 and Ser 1184) that cleave nsp3 to nsp12 [33,34]. nsp9 is a highly conserved RNA-dependent RNA polymerase, whereas nsp10 is a helicase that presents a metal-complexing region at its amino terminus [34]. The functions of proteins nsp11 and nsp12 remain unknown, though has been reported that nsp11 shows specific pyrimidine endo-ribonuclease activity as well as a conserved domain present in all nidoviruses [35]. It has been suggested that the nsp3, nsp5, nsp6, nsp7, and nsp8 proteins are responsible for the virulence of PRRSV [14]. Recently, Li *et al.* found that the nsp9 and nsp10 increase the virulence of the atypical HP-PRRSV emerging in China [36], but information regarding mechanisms by which they could contribute to pathogenicity remain unknown.

## 3. Modulation of Innate Immune Responses by Non-Structural Proteins

Type I interferons are key elements of the innate immune response involved in viral infections. Among their functions include: (a) the generation of an antiviral state in cells that blocks viral replication; (b) the promotion of the development of CD8^+^ T cells; (c) the regulation of MHC-II (major histocompatibility complex); (d) the promotion of the differentiation of antigen-presenting cells; (e) the stimulation of the proliferation of memory cells through IL-15; and (f) the prolongation of the half live of activated T lymphocytes [37,38].

Many viruses [39,40,41], including PRRSV [42,43,44,45], have mechanisms for inhibiting the production of interferons to evade the immune system. PRRSV has been shown to be a poor IFN inducer in *in vitro* models of macrophages [46]. The inhibition of type I IFNs has been shown to be strain dependent for plasmacytoid dendritic cells, whereas inhibition of IFN-α and IFN-β appears to be more general in myeloid DCs, macrophages and non-macrophage cell lines such as MARC-145 and BHK-21 [47,48].

The inhibition of type I IFNs has been largely attributed to the ability of nsps to inhibit the promoter of IFN-β by blocking the translocation of IRF3 and the inhibition of NF-kappaB (NF-κB), though other mechanisms are thought to exist. As shown by Calzada-Nova *et al.* a UV-inactivated virus retains the ability to inhibit IFN-α responses of plasmacytoid DCs, a fact that indicates that inhibition may also be related to the interaction of PRRSV with the surface receptors of plasmacytoid DCs [49]. However, in addition to a decrease in IFN expression, the lack of IFNs affects the expression of other proteins important to anti-viral response, including interferon-stimulated genes (ISGs), such as the protein produced by the gene ISG15 [50]. It has been observed that in contrast to what occurs with the classical genotype 2 North American isolates, neither protein kinase R (PKR), 2’,5’-oligoadenylate synthetase (OAS) or MX are induced in highly virulent PRRSV infection, suggesting that the production of IFN is decreased by the down-regulation of ISG [51].

Recent studies have shown the participation of individual PRRSV proteins in IFN inhibition. nsp1 (both subunits α and β) has been reported to be one of the main elements involved in the inhibition of type I IFNs [52,53,54]. nsp1 has been shown to suppress the activity of NF-κB by inhibiting IκBα phosphorylation, resulting in the down-regulation of the IFN promoter [55,56]. Interestingly, this ability is strongly mediated by the C-terminus of nsp1α. In fact, the inhibitory potential is lost when this region is deleted [55]. Additionally, nsp1α degrades CREB-binding protein, preventing the recruitment of IRF3 for enhanceosome assembly and consequently blocking IFN induction [57,58] (Table 1).

**Table 1:** Inhibition of type I IFNs is the result of multiple nsps and mechanisms*.

nsp	Cell Tested	Mechanism	Reference
**nsp1**	MARC-145, HEK-293T	↓ phosphorylation of IRF3	[52,56]
	MARC-145, HeLa	Degradation of CREB-binding protein	[57]
	HeLa	↓ IFN-β promoter activation	[52]
**nsp1α**	HEK-293, HT1080	↓ IRF3-mediated gene activation	[52]
	HEK-293T	↓ IFN-β promoter activation	[53]
	HeLa	↓ IκB phosphorylation and nuclear translocation	[55]
**nsp1β**	HEK-293, HT1080	↓ IRF3-mediated gene activation	[52]
	HEK-293T	No effect on IRF3	[53]
**nsp2**	HEK-293T	↓ function as a deubiquitinating enzyme	[59]
		↓ phosphorylation of IRF3	[60]
	HeLa	↓ IFN-β promoter activation	[52]
**nsp4**	HeLa, MARC-145	↓ IFN-β promoter activation	[52,61,62]
**nsp11**	HEK-293, HT1080, MARC-145	↓ IRF3-mediated gene activation	[52,63]
	HeLa, MARC-145	↓ IFN-β promoter activation	[52,63]

* Adapted from [44].

The central region of nsp2 has been shown to play an important role in the regulation of the innate immune response, and the protein contains several linear immunodominant B-epitopes designated ES2 to ES7 [32]. Mutant viruses harboring a deletion in the region corresponding to ES3 (located in the central hypervariable region) have an increased cytolytic activity and replicate to higher titers than the parental strain. This situation correlated with a decreased potential for inducing IL-1β and TNF-α release by infected macrophages, an indication that nsp2 is involved in the regulation of the innate response and most likely in attenuation/virulence [64]. Recently, it has been shown that nsp2 indeed does contain different domains with several functions. In HeLa cells, nsp2 may activate the NF-κB pathway; because this was related to the hypervariable region of nsp2 [65], the effect could be different depending on the viral strain. In addition, the N-terminal PLP2 domain of PRRSV nsp2 is capable of inhibiting the ubiquitination of retinoic acid-inducible gene 1-a pattern recognition receptor, which binds double-stranded RNA, interfering with the innate response of the cell [59,66].

Chen *et al.* found that nsp4 of the highly pathogenic PRRSV (HP-PRRSV) strain JXwn06 has severe inhibitory effects on IFN-β, NF-κB and IRF3 compared to the low pathogenic PRRSV strain HB-1/3.9. These effects were demonstrated with a change in aminoacids at residue 155 (Thr to Lys); this change improved nuclear localization and inhibition of IFN-β transcription by nsp4. This study suggests that the nuclear localization of nsp4 could have an important role in the viral replication of PRRSV, besides its characteristic serine protease activity in the cytoplasm [61,67]. A recent report describes that nsp4 suppress IFN-β expression through the interference of NF-κB signaling pathway by targeting the NF-κB essential modulator (NEMO), suggesting that targeting NEMO could be an effective strategy for viruses to inhibit the NF-κB pathway [62].

In EAV, Endoribonuclease (NendoU) activity has three catalytic residues (Hys-126, Hys-146 and Lys-170) [35], in PRRSV, this site is located at residues Hys-129, Hys-144 and Lys-173. In a mutagenesis assays performed by Shi *et al.* the deletion on Hys-129 disrupts this activity and also loses the ability to block the IFN-β promoter [54]. In agreement with these results, Sun *et al.* demonstrated that the PRRSV nsp11 was able to inhibit the IFN-β expression in a dose-dependent way on MARC-145 cells [63].

Information on other nsps involved in the regulation of the innate immune response is scarce. Recently, Han *et al.* [68] demonstrated that nsp1 subunits of others arteriviruses such as EAV, LDV and SHFV, are able to modulate IFN production by inhibiting IFN promoters or blocking signaling pathways (IRF3 and NF-κB). As it is plausible that there may be other regions of PRRSV nsps involved in the positive or negative regulation of the immune response, it is essential to identify the viral proteins and their domains that have the capacity to inhibit innate responses in an effort to understand how the virus may subvert the innate immune response and to develop effective vaccines.

## 4. Humoral Immune Response to Non-Structural Proteins

In the course of PRRSV infection, antibodies develop rapidly, mainly against the N protein; however, these early antibodies are not protective and may also contribute to the phenomenon of antibody-dependent enhancement (ADE) [69], though ADE has not been confirmed in the field. Neutralizing antibodies appear later and are thought to be directed principally against GP5, GP4, GP3 and most likely GP2 [70,71,72,73]. Most studies pursuing antibodies directed at nsps have focused on the potential for differentiating between strains, either for vaccination or for differentiation between genotypes.

Using a phage display system, Oleksiewicz *et al.* showed that linear B-epitopes could be found in nsp1, nsp2, and nsp4, and the nsp2 epitopes were considered to be immunodominant (Table 2) [32]. Subsequently, de Lima *et al.* prepared a collection of 97 overlapping synthetic peptides from nsp2 and tested them with sera from PRRSV hyperimmune pigs [31]. Their results showed that 10 of the 97 nsp2 peptides were recognized by more than 80% of the sera from pigs that had been infected for up to 90 days with the North American-type NVSL 97-7895. The majority of those peptides contained regions rich in hydrophilic amino acids which may enhance the recognition by B-lymphocytes [31], and some of them were located in non-essential regions of nsp2 that could be deleted without affecting the viability of the virus, thus having the potential to be used for differential diagnosis [74]. Unfortunately, several reports indicate that deletions in nsp2 are very frequent in wild-type PRRSV, compromising the use of this protein as a marker for differential vaccines [13,20,21,22].

**Table 2:** Role of nsps in the immune response.

nsp	Humoral response	Cellular response	Reference
nsp1	√		[75]
	√		[76]
	√		[32]
		√	[77]
nsp2	√		[75]
	√		[31]
	√		[76]
	√		[32]
		√	[77]
nsp4	√		[76]
	√		[32]
nsp5		√	[78]
		√	[77]
nsp7	√		[75]
		√	[77]
nsp9		√	[79]
		√	[77]
nsp10		√	[79]
nsp11		√	[78]

Other authors have described similar results for recombinant nsp1 and nsp2 [76] and showed that recombinant nsp1 and nsp2 could be recognized by sera obtained from pigs infected with different PRRSV strains. Although it was not possible to identify conserved regions, a highly variable region was located, suggesting that this region could be a marker for distinguishing between vaccinated and infected animals.

At the initial stages of infection, the kinetics of the humoral response against nsp2 are similar to that of the N protein, though in the long term, antibodies against nsp2 persist for longer than anti-N antibodies [80]. Memory B-cells producing anti-nsp antibodies are found mainly in the tonsils during the persistent stages of the infection [80]. Brown *et al.* [75] evaluated the humoral response to nsp1, nsp2 and nsp7 proposing that nsp7 is a good candidate for diagnosis and could be evaluated as a marker for differentiating infections of genotypes 1 and 2.

The humoral response to other arterivirus nsps is scarce. Go *et al.* [81] expressed recombinant nsp1 to nsp12 and evaluated the immune response by immuneprecipitation using serum samples of experimental infected horses with different strains (n=3), persistently infected horses (n=3) and vaccinated horses with a modified live vaccine (n=4). Results showed that nsp2, nsp4, nsp5 and nsp12 were the most immunogenic nsps of EAV. nsp1, nsp3, nsp6 and nsp11 were not immunoprecipitated by the serum samples. It is interesting to remark that all serum samples contained VN antibodies titers. These data suggest a differential humoral response against EAV nsps as observed in PRRSV nsps.

## 5. Cell-Mediated Immune Response to Non-Structural Proteins

It is generally accepted that PRRSV induces an unusual adaptive immune response, with a delayed development of neutralizing antibodies and the cell-mediated response [82,83], as previously reviewed [84], and this response is thought to be largely caused by the ability of the virus to modulate the immune response. However, it is generally accepted that once the virus is cleared and immunity develops in full, immunity against a homologous re-infection is generally sterilizing, whereas protection against a heterologous strain is only partial.

The participation of nsps in the development of the cell-mediated immune response has received comparatively little attention. A study by Parida *et al.* describes peptides present in nsp9 and nsp10 as capable of inducing effector/recall IFN-γ responses [79]. In that study, the authors synthesized 17-mer peptides from both proteins (nsp9 and nsp10 of PRRSV genotype 2 strain FL-12) to determine their capacity for inducing the proliferation of T cells. Initially, they identified groups of reactive peptides that were then tested individually; those that retained their capacity for stimulating proliferation were tested to determine their capacity to induce the production of IFN-γ. Finally, four peptides from each protein were identified as having the capacity to induce both a proliferative response and a response for IFN-γ; three of the four peptides were found in conserved regions of the proteins. In another study that used bioinformatics to predict T-cell epitopes from conserved regions of PRRSV genotype 1 and 2 followed by *in vitro* confirmation, the authors described peptides from nsp2 and nsp5 capable of inducing IFN-γ responses [78]. Recent results from our laboratory also indicate that certain peptides from nsp2, nsp5, nsp9, and nsp11 are able to induce IL-10 [78] in recall responses. As described by Burgara-Estrella *et al.* [78] some of the IL-10-inducing peptides were able to inhibit the production of IFN-γ induced by PHA [78]. These results suggest that the capacity to modulate the immune response through IL-10 is an intrinsic property of some nsps of PRRSV. This redundancy in induction represents a complicated challenge that remains to be resolved (Table 2).

Recently, a study carried out by Mokhtar *et al.* [77] reported a synthetic overlapping peptide library representing the 19 proteins of PRRSV genotype 1 composed of 1276 15-mer peptides. The aim of that study was to elucidate the specificity of the T cell response induced by PRRSV 1 infection, as well as to determine the phenotype of the T cells responding to a given peptide. Mokhtar *et al.* [77] found T cells that produced IFN-γ when stimulated with peptides from nsp1, nsp2, nsp7, nsp9 and nsp11 as well as other peptides from N, M and GP3 structural proteins. The above response was observed in two of three pigs repeatedly infected with PRRSV; despite the limited number of pigs, the data indicate a marked diversity in the response to nsps, which is most likely attributable to the MHC haplotype. However, the results also demonstrate that the conserved regions in nsps (such as nsp1β_149–163_) can be highly antigenic. In this same study, the authors tested two additional groups of pigs infected with two different PRRSV strains. Those pigs that were infected with PRRSV strains with closely related peptide sequences showed a significant response to nsp1β and nsp2 peptides, and this response was similar to that in pigs infected with divergent PRRSV strains [77]. Therefore, the similarities of responses among divergent strains can be attributed to the rate of conservation of the peptides between divergent strains. It is important to mention that Mokhtar *et al.* and Parida *et al.* used overlapping peptides, in contrast, Burgara-Estrella *et al.* used bioinformatics tools to predict T-cell epitopes. Despite this discrepancy, the above studies revealed the participation of nsps in the induction of IFN-γ. Further studies will reveal which nsps peptides or nsps are able to induce protective immunity against PRRSV.

## 6. Conclusions

Due to the importance of PRRSV in the swine industry, much information about the mechanisms of regulation has been reported. Although the current knowledge of these mechanisms is insufficient to completely understand the immuno-pathogenesis of the virus, recent studies have focused on the non-structural proteins involved in modulating and inducing the immune response. Scarce information about nsps regarding the cellular response has been reported; however, studies demonstrate that nsps contain regions that are highly antigenic and conserved between different PRRSV strains, suggesting that the utilization of some nsps as a target of the immune response is more important than initially thought. In the future, vaccines based on conserved immunogenic nsps could induce protection against different strains, with an effective and rapid response to infection and preventing the immuno-modulatory effects of the virus. Within this scenario, nsps could be good candidates for in-depth exploration.
